# Learning to prescribe and instruct exercise in physiotherapy education through authentic continuous assessment and rubrics

**DOI:** 10.1186/s12909-020-02163-9

**Published:** 2020-08-08

**Authors:** Doris Yin Kei Chong, Barbara Tam, Suk Yu Yau, Arnold Yu Lok Wong

**Affiliations:** 1 Department of Rehabilitation Sciences, The Hong Kong Polytechnic University, Hong Kong SAR, China; 2 The Hong Kong Society for Rehabilitation, Hong Kong SAR, China; 3 Educational Development Centre, The Hong Kong Polytechnic University, Hong Kong SAR, China

**Keywords:** Authentic assessment, Rubrics, Physiotherapy education, Self-regulated learning

## Abstract

**Background:**

Authentic assessment and effective feedback are among various strategies that promote learning in the assessment process. These strategies are commonly used during clinical placements. However, they are rarely implemented in the didactic portion of physiotherapy education despite the benefits this type of assessment may bring to achieving students’ learning and outcome.

**Methods:**

This mixed method study investigated how an authentic continuous assessment coupled with rubrics facilitated physiotherapy students’ learning process in a real-life complex skill of exercise prescription and instruction. The study also explored the relationship between different activities in the Learning Management System (LMS) and learning outcomes. Qualitative data was collected using a focus group and an analysis of discussion forum posts. Quantitative data included various information from a questionnaire, the LMS and assessment score.

**Results:**

Thematic analyses from the focus group and discussion forum posts suggest that students used a cyclical self-regulated learning process as a result of authentic task design and rubrics for feedback facilitation. Interestingly, the discussion forum access was found to be moderately and significantly correlated with assessment score by Spearman’s rank correlation (ρ = 0.59, *p* < 0.01), while the students did not find the discussion forum useful.

**Conclusions:**

Overall results suggest the promotion of self-regulated learning in this authentic continuous assessment. The roles and goals of each authentic task within the assessment should be made explicit in order to raise cognitive awareness of benefits.

## Background

Formative assessment has been widely promoted in education [1] to counteract the overreliance of summative assessment. It has achieved popularity and beneficial effects in various educational fields including health care education [2, 3]. However, the argument remains on the need to further increase the proportion of formative assessment over summative since formative assessment can be deemed to be associated with learning through the feedback and reflective process [4, 5]. In recent years, formative assessment is often situated in a broader assessment concept termed Assessment for Learning (AfL). Both concepts stress the importance of using feedback to facilitate learning [6, 7]. The key principle in AfL is using assessments to facilitate learning rather than only evaluating the outcome of learning. However, it does not mean to exclude the evaluation function altogether but to focus more on the learning function in assessment. Carless [8] advocated four strategies to actualise and scale up AfL: assessment task design, effective feedback processes, discernment and judgement of quality of work. The first strategy is to design assessment tasks that align with learning objectives and mirror real-life situations. When this assessment design is used, it is termed authentic assessment.

In physiotherapy education, clinical placement is a classic example of authentic assessment, where students learn to examine and treat clients in real clinical settings. Students are being assessed continuously during the placement period that usually lasts for weeks. However, this type of authentic continuous assessment in the didactic component of the curriculum rarely exists. Limited evidence has shown that pre-clinical authentic assessment enhances collaboration and negotiation, aids learning and reduces stress [9, 10]. Yet, previous studies included only a one-time rather than continuous assessment.

One example of real-life skills in the field of physiotherapy that may benefit from continuous authentic assessment in the didactic portion of physiotherapy education is physical activity and exercise prescription. Incontestable evidence has shown the importance of physical activity and exercise in preventing or managing the adverse effects of chronic diseases [11]. In response to the worldwide needs for promoting and implementing healthy life style through physical activity, the international professional body of physiotherapy, the World Confederation of Physical Therapy, recently renamed as the World Physiotherapy, issued a position statement that stipulated ‘physiotherapists as exercise experts’ to deliver care through health promotion and wellness [12]. To uphold this standard, it is crucial for physiotherapy graduates to possess adequate knowledge in exercise principles and prescription, as well as to be able to integrate and apply this knowledge in real-life situations. Entry-level physiotherapy programmes are no doubt in the position to ensure this standard [13]; hence, there are tremendous opportunities to implement learning activities and assessments to match the requirements.

This study investigated students’ experience in a six-week authentic assessment of exercise prescription and instruction. This redesigned assessment format is necessary because students simulated the role of a physiotherapist during the six-week period and they experienced the role as a clinician before clinical placement. We believe that this experience is authentic according to Carless’ definition [8] and can help students engage in the skills required in real life clinical situations. However, this experience is rarely provided to students in the didactic portion of the physiotherapy curriculum, and highlights a gap in the existing assessment practice. Thus, the main aim of this study was to investigate the perceived benefits of this authentic continuous assessment to facilitate students’ learning, and to explore how learning could be achieved.

A secondary aim of this study was to explore the relationship between usage patterns in the learning management system (LMS) and learning outcomes as measured by the course grade. This authentic continuous assessment process included the use of rubrics housed in the LMS. Rubrics were used as a grading and feedback tool because they have been shown to improve transparency of grading criteria and thus facilitate students’ self-assessment of performance [14, 15]. Rubrics not only let students know their grade but also guide their learning [16]. It is therefore reasonable to assume that usage patterns in the LMS correlate with learning outcomes. The benefits of authentic assessment and rubrics in AfL are explained in the following sections.

### Benefits of authentic assessment

Authentic assessment task design should be the first thing to consider in order to set up an AfL environment [6, 8]. The key ingredients in designing an authentic assessment are a real-life context and meaningfulness. In other words, assessment tasks need to match with what students will encounter in their respective industry in real work situations. Another important arena of authenticity is the degree of complexity embedded in the assessment task. The task should be complex enough where students are required to solve problems as if they are facing a real-life issue [6]. Through this type of “training” in authentic assessments, students could build up the abilities required for complex situations beyond life in higher education and therefore produce the attribute of lifelong learning [17].

The benefits of authentic assessments in higher education have been reported in the literature. They increase students’ satisfaction and positively promote behaviors essential for the field [18]. If the task involves group work, it can build team effort and confidence [19]. Since an important aspect of authentic assessment is stressing appropriate task complexity, problem-solving skills are also enhanced through authentic assessment task design [20].

However, it is not very clear how authentic assessment leads to the abovementioned benefits, when the bigger concept of formative assessment is reviewed, it is found to link with self-regulated learning [15] and this may be the mechanism behind the benefits of authentic assessment. Self-regulated learning contains three cyclical phases: forethought, performance and self-reflection [21]. The forethought phase includes elements such as planning and happens before the actualisation of tasks. The performance phase means actualisation and action. The self-reflection phase happens after the fact and includes comparisons between expectation and the actual performance. The nature of formative assessment can provide the context and processes (e.g. feedback to be used for improvement before a final grade is confirmed) needed to support all phases of self-regulated learning [22]. Effective feedback is also one of the strategies mentioned by Carless [8] in order to scale-up AfL. It is evident that the elements in AfL or formative assessment and self-regulated learning are intertwined to optimise learning during the assessment process.

### Benefits of rubrics in AfL

At its simplest level, a rubric is a tool to guide scoring of students’ performance [23]. A rubric should provide a qualitative description of discrete evaluative criteria, and each criterion should have a respective score or a grade for differentiation [23]. In recent years, there has been a stress on using rubrics as a learning or instructional tool in addition to just a scoring tool. Rubrics can, and should, serve both summative and formative functions to: (1) provide students and teachers with essential information on their overall performance; and (2) be used as a formative way for students to improve and learn [24, 25].

Numerous benefits of rubrics have been reported in the literature. Because rubrics spell out assessment criteria in an organised format, they increase transparency, grading consistency and students’ understanding of expectations [14, 26, 27]. These benefits may mediate students’ learning, in identifying goals, focusing on quality work, facilitating work collaboration, and reducing anxiety during the assessment process [24, 27, 28]. If rubrics are available to students at the beginning of the assessment, they can be served as a planning tool to support the forethought phase in self-regulated learning, and to assist them in planning how they can reach the end goal of the assessment [22].

Another benefit of rubrics lies in their use as a feedback tool. It has been shown that students reflect on feedback from rubrics for planning of future work [24, 26]. This is the reason rubrics are used primarily in formative assessment, as they provide information in an explicit and organised manner for students to reflect on before final submission of assessment. Self-reflection is also a key phase in self-regulated learning; hence, rubrics, if used appropriately, may support self-regulated learning in the assessment process. However, it remains a challenge to fully actualise the learning benefits of rubrics, in that it requires a holistic implementation and literacy of all stakeholders involved [29].

### Aims of this study

The primary aim of this study was to explore students’ perceived learning benefits and their learning processes in this authentic continuous assessment. The secondary aim was to investigate the usage patterns of various LMS activities and their relationships with the learning outcome (i.e. assessment scores). The qualitative data was primarily used to address the primary aim; however all types of data were interpreted together to obtain a full picture of the research questions. Although the study intended to explore how students learnt, there was no pre-set hypotheses of learning processes. The process would be identified through inductive reasoning during data analysis.

## Methods

### Research context

This study involved a cohort of Year-2 students in their first semester of the undergraduate physiotherapy curriculum in one university in Hong Kong. Students were taking five mandatory courses. They were exercise science, pharmacology, orthopaedics and traumatology, human physiological changes across lifespan, and principles of physiotherapy practice. These courses do not consist of a practical component except for the course, principles of physiotherapy practice, where students were exposed to basic skills and were assessed in one-off examinations. The “intervention” authentic continuous assessment is an assessment component within the course, exercise science. For this assessment, students exercised together in pairs for a total of 6 weeks. There was a 1 week school holiday in addition to the six-week period. During this period, each of them played the role of an exercise instructor and a client. Students conducted a baseline physical fitness examination (e.g., fat composition, height, weight, cardiovascular fitness, flexibility, as well as trunk, upper limb and lower limb muscle strengths) on each other in the first laboratory session of the course, identified exercise needs of their partner, set goals, and submitted a preliminary exercise plan to the lecturers for feedback before proceeding with any exercises. Each week, they were required to submit a weekly log and a communication video on the LMS to report on exercise progress. The purpose of communication videos was to allow lecturers to understand how exercise instruction was delivered, as this is an important aspect of exercise prescription. Students were also required to participate in the graded discussion forum to discuss and seek advice on issues (from peers and lecturers) they faced during the exercise period.

**Fig. 1:**
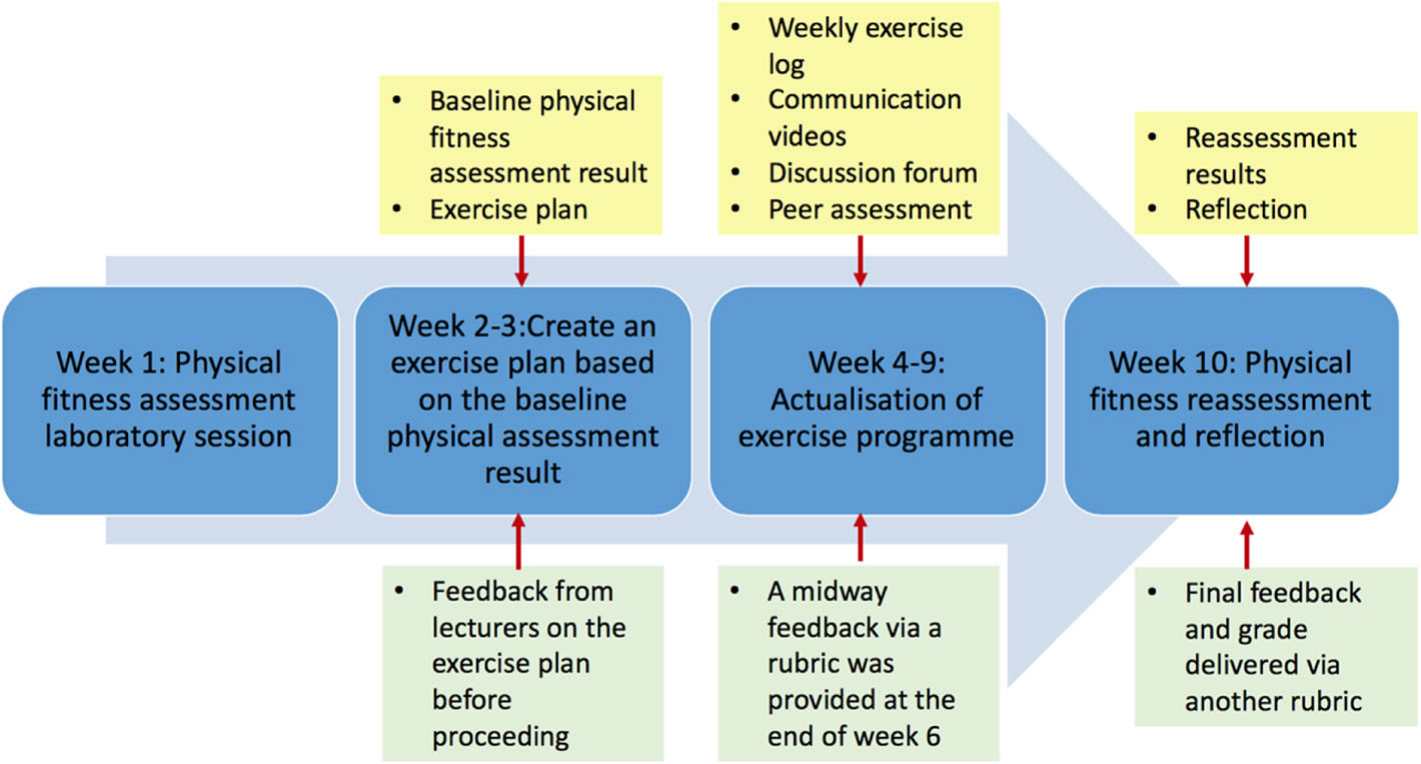
Assessment activities, tasks and feedback timeline. *Blue boxes indicate assessment activities. Yellow boxes indicate submission components. Green boxes indicate feedback details and time

Lecturers graded various assessment tasks and provided feedback to students at three time points (at the beginning, mid-point and on completion) using rubrics available on the LMS from the first day of class. In addition, the rubrics were explained to students in the first class who were reminded to refer to them throughout the assessment period. A peer assessment rubric on communication skills was also included. This way, students could make use of rubrics and feedback to continuously modify their exercise instruction and prescription, in order to reach learning objectives of this assessment. Figure 1 illustrates the assessment tasks and grading/feedback timeline.

### Study design, sample and data collection

In order to understand how students learnt and utilised the rubrics, as well as the effects of rubrics and/or other LMS contents on learning outcomes, this study employed a mixed methods approach to collect and evaluate various data sources. A focus group and a questionnaire (Table 1) were used to understand students’ perceptions on this assessment, rubrics and the LMS experience. The content of the discussion forum was analysed to explore students’ learning process in this assessment. In addition, LMS access data and the final scores of this assessment were used to analyse the LMS utilisation pattern and its relationship with the learning outcome. The study was funded by the Teaching Development Grant (2016–2019) of the university. Ethics approval was obtained from the Departmental Research Committee (Reference number HSEARS20180105004) prior to the data collection.

**Table 1:** Questionnaire items and responses

Item	Percentage of positive responses		
	Agree	Strongly agree	Total positive
1. Exercise prescription assignment provided me an opportunity to practice exercise instruction as a therapist.	64%	23%	87%
2. I am clear about the objectives of the exercise prescription assignment and the expectation in each stage of the learning process.	63%	11%	74%
3. Exercise prescription assignment has increased my confidence in critically analysing situations relevant to exercise progression.	53%	11%	64%
4. Exercise prescription assignment has strengthened my ability to apply and integrate exercise science knowledge.	72%	12%	84%
5. Exercise prescription assignment has enhanced my ability to reflect on my learning strengths and weaknesses.	57%	10%	67%
6. Exercise prescription assignment has improved my ability to provide exercise training advice to individuals with different needs.	66%	15%	81%
7. The online discussion forum facilitated my communication with peers and instructors throughout the exercise process.	28%	7%	35%
8. The Blackboard site (e.g. Timeline) was useful for me to track progress.	53%	26%	79%
9. The rubrics provided clear criteria for each grade level.	54%	10%	64%
10. The rubrics increased my understanding of the expectations of the exercise prescription assignment.	57%	9%	66%
11. The rubric language was appropriate for me to understand.	61%	18%	79%
12. The feedback and guiding questions from instructors and peers facilitated me to think what I have been doing and how the process can be improved.	65%	11%	76%
13. Accessing information and feedback anytime and anywhere on Blackboard facilitated my learning.	66%	15%	81%
14. The peer and instructor feedback improved my exercise prescription and instruction skills throughout the process.	66%	15%	81%

Six students were purposefully invited to participate in the focus group, according to their willingness to share. According to Krueger & Casey [30], six to eight participants in a focus group is generally an accepted number in applied research. Five students accepted the invitation and we were unable to recruit another participant before the date of the focus group. Therefore, it was conducted with five participants. Nyumba, Wilson, Derrick and Mukherjee [31] quoted in their paper that a group size of four to fifteen participants was used in previous research and was deemed sufficient. Therefore, for the nature of this study, we conducted the focus group with five participants. In addition, since focus group data was only one of the data sources in our study, we felt that it is acceptable to continue with the five participants. The focus group discussion was conducted in a private room 2 weeks after the authentic continuous assessment was completed. The discussion was conducted in Cantonese, lasted for about 70 min and was audio recorded.

In addition, all students enrolled in the course (*n* = 117) were invited to participate in an online questionnaire. The questionnaire consisted of 14 items exploring their experience regarding the authentic continuous assessment, rubrics and LMS. They rated their responses to each item on a 5-point Likert Scale where 1 indicates strongly disagree and 5 means strongly agree. There was one open-ended question at the end of the questionnaire to collect additional comments. Access patterns of the rubrics and discussion forum were downloaded from the LMS after the end of the semester for data analysis. Discussion forum content was also downloaded and reviewed. There are numerous data available in the LMS; for the interest of this study, only the number of hits of various content areas in the LMS and their relationships with assessment grades were investigated. The time-period for counting the number of hits was from the first day of class to the due day of the final component of this assessment.

Various data collection methods were used to evaluate the effects and understand the perceptions of authentic continuous assessments on the learning outcome. The focus group allowed the exploration of in-depth meanings of students’ perceptions and experience in this authentic continuous assessment experience. Since the participants were from the same cohort and shared similar experience, it was deemed appropriate to facilitate an interactive group dynamic to generate fruitful discussion that may otherwise be impossible [32]. All research that relies on self-reports are subject to the problems of informant inaccuracy. This research used cued recall and aided recall [33] to ensure accuracy and preciseness of the narratives from the focus group discussions to recall learning behaviours. In addition, in order to maximize the effectiveness of the focus group discussions, the facilitator also asked questions that were non-sensitive so that participants would feel at ease and indicated their responses would be valued so that participants would be willing to answer those questions [34].

Analysing discussion forum posts allowed the researcher to understand more in-depth what had actually happened during the authentic assessment period, including, but not limited to, the challenges they faced and how they solved the issues. On the other hand, the questionnaire allowed more students to provide their views on the perceived usefulness of different aspects of this authentic continuous assessment. Importantly, the questionnaire, LMS and assessment scores are more concrete data that can supplement the results gathered from the focus group and discussion forum. Hence, a broader representation and understanding of students’ experience and its effect would be available.

### Data analysis

Focus group audio data was translated and transcribed into English first by a project assistant and cross-checked by the first author. Focus group and discussion forum content data were analysed separately using thematic analysis by the first author. Thematic analysis based on the six phases by Braun and Clarke [35] was adopted: (1) become familiar with the data; (2) generate initial codes; (3) search for themes; (4) review themes; (5) define themes; and (6) write-up. Qualitative data from the focus group and discussion forum posts supplemented each other and reach data saturation through triangulation [36]. As only the first author was involved in qualitative data analysis, reflexivity was constantly exercised to remind and reflect on the role as a researcher and to locate any personal bias [37]. For example, the first author’s epistemological belief that knowledge is constructed through both personal/social experience and objective facts may lead the data analysis towards this direction. Being aware of this personal belief minimises bias from happening. In addition, the authors read the themes and agreed on the analysis. This served to safeguard bias and enhanced trustworthiness of data.

For the questionnaire, the combined percentage of ‘agree’ and ‘strongly agree’ was reported and was considered as favourable results. The Statistical Package for the Social Sciences version 25 (IBM Corp, Armonk, NY) was used for all statistical analyses. Descriptive data from the LMS and assessment scores were reported.

Since all students needed to access relevant teaching materials (e.g., online lectures, lecture and tutorial notes, rubrics, and discussion forum) and to submit their assignments through the LMS, the course worked on the premise that students were required to access the LMS regularly. The duration and/or frequency students spent on accessing the LMS was hypothesised to correlate with their efforts, and in turn better assessment scores. While this method was not validated in this current study, it has been used elsewhere in other studies where the authors correlated access data and performance scores [38–40].

The correlations between various LMS access data and the assessment score were evaluated by Spearman’s rank correlation coefficients because the data were not normally distributed. The correlation coefficient (ρ) was interpreted as weak (0.30 to 0.50), moderate (0.50 to 0.70) and strong (0.70 to 0.90) based on the literature [41]. The significant level was set at 0.05. All qualitative and quantitative data were triangulated and interpreted by all authors to get a well-rounded picture of students’ learning experience and process in this assessment.

## Results

The results are reported in four sections: (1) thematic analysis from the focus group interview; (2) thematic analysis from the discussion forum; (3) quantitative findings from the student questionnaire; and (4) quantitative findings from the LMS data and correlation analysis of assessment scores. In particular, results from the focus group analysis and the discussion forum analysis explored if and how students learnt in this authentic continuous assessment. Results from the focus group analysis, questionnaire, LMS data and correlation analysis explored students’ use of rubrics and other LMS content areas, as well as their relationship with learning outcomes.

### Focus group analysis

Two themes related to the study objectives were identified from the focus group analysis. The first theme was ‘learning through self-regulation and reflection in authentic assessment’ while the second theme was ‘guidance and feedback with rubrics’. The two themes are further elaborated below.

### Learning through self-regulation and reflection in authentic assessment

Focus group participants regarded this authentic continuous assessment as a new and habit changing experience. Although they questioned the authenticity of some of the assessment tasks, e.g. communication videos and discussion threads, in the forum, they thought that it was overall a good and different exposure. They shared the view that in this assessment, they needed to think through the process beforehand, learn through mistakes and peer interaction, and reflect through watching their own videos. The entire assessment process mirrored a real-life scenario which was beneficial to their learning.

‘I think it is good for learning because during the filming and planning process, we tried to *predict* what kind of difficulties my partner may experience and then the whole process made us aware of the actual situation of a real patient.’ (S1)

‘It is a good experience because the opportunity of prescribing the whole exercise plan is rare. I did something similar before for myself but it wasn’t this detailed. But this assessment helped us *think* more thoroughly.’ (S3)

‘ … The assessment is good because it involves both written and practical components into an assessment, even for watching the videos, we could *see what we did well and bad (and modify)*.’ (S4)

These quotes suggest that students regulated their own learning experience in this assessment through predicting, thinking and modifying. These actions illustrate students work from the planning phase to reflecting on their performance afterwards. Since this was not a one-off assessment, students needed to continuously reflect on and modify their performance in order to reach the end goals, be it just finishing the assessment or finishing it with a high mark. The nature of this assessment requires them to utilise different strategies (planning, reflection, etc.) in order to fulfil objectives, a process required in real-life situations within the physiotherapy practice.

This self-regulated process appeared to have continued after the midway feedback, in which students used a different approach to address the second half of the assignment as a result of the midterm score:

‘ … but after knowing the (midterm) score, there appeared to have substantial differences between the first and last 3 weeks … we no longer just bound by the lecture requirement and would ask for his (assignment partner) feeling on the (exercise) experience … he would suggest what he really wants to do … we could really contribute to a better relationship to encourage each other to exercise.’ (S2)

Although the student did not explicitly state a second round of self-regulated process, the quote suggests that the student self-reflected on his work in the first half (as a result of his score), then modified his work for the second half of the assessment process to focus on building rapport with his “client”. The strategy used in the second half was completely different. It is reasonable to state that a cyclical self-regulated process was used in this scenario.

### Guidance and feedback with rubrics

Participants’ experience with rubrics, in this assessment, included using them to guide their work and to understand teachers’ expectations. They viewed rubrics as a standard requirement they need to follow, though which may limit creativity at times.

‘Rubric itself is good because for example, the video taking, we wouldn’t know what to expect and include in the video, but rubrics help us know what to expect.’ (S3)

‘I think rubric is like a guideline … we would feel frustrated over not knowing what to do … without specific guideline, we don’t know what to do.’ (S4)

Despite providing guidance and setting expectations, participants wanted to see a more flexible approach with rubrics to allow for creativity when appropriate. They shared the view that rubrics are too “standardised” and students are scared not to follow them, thus this may limit their creativity if they want to submit something not included in the rubrics.

‘ … having a rubric is better than none as you can at least tell what teacher is expecting but that is not the most ideal … everything is standardised.’ (S2)

‘ … the grade difference is huge when we do not follow the guideline (rubric), nobody would dare not to follow … it should encourage us to try our own approach.’ (S1)

These quotes resonate with the inherent limitations of rubrics where higher level creativity may be limited [42]. Nonetheless, the rubrics in this assessment appeared to serve their functions to guide learning for students. In addition, students also made use of the grade descriptors in rubrics as feedback to guide subsequent submissions.

‘ … seeing the midterm grade (in rubric) is good, you would know if you are doing it reasonably, so you would feel less anxious about subsequent work.’ (S4)

In fact, students appeared to demand more frequent feedback from rubrics in order to guide their paths of reaching assessment objectives:

‘Actually, among all assessment (except practical), this (assessment) is actually quite good, coz it’s ongoing with feedback. I like feedback because we often don’t have feedback and we don’t know which parts we did wrong. But one thing that I think can improve is that, perhaps some feedback at the beginning, then another round of feedback few weeks later, is quite okay. It is better not to just give feedback once in midway and at the end because that would be quite inconsistent and not that good.’ (S5)

These quotes illustrate that rubrics do not only guide the planning phase of assessment but also subsequent works. Students make use of feedback (in grade descriptors in rubrics) to modify their work for future improvement. This process is self-regulated, with the rubrics being a tool to facilitate this process.

### Discussion forum analysis

Discussion forum posts by students were read, to draw themes on learning processes in this authentic continuous assessment. The results supplement themes identified from the focus group interview, and help understand how students applied and integrated exercise prescription knowledge in this assessment. Analysis revealed one central theme that was similar to the first theme from the focus group analysis: learning through self-regulated procedures. These procedures include thinking and planning, searching, assuming, trialling, listening, discussing, reflecting and modifying.

Based on the analysis of the discussion posts, it was clear that before students embarked on the assessment, they thought through the steps they needed to do to come up with an exercise plan, and how they could actualise the plan, including a feasible schedule and environment. In addition to thinking, they actively searched for information (lecture notes, textbooks, online materials) on how to prepare an exercise plan. They added some assumptions based on their own knowledge and experience on how their plans might work. These were all considered the planning phase of this learning process.

The doing phase involves trial and error of their plans. When things did not occur according to plan, they discussed among themselves and listened to each other for feedback.

‘I have planned to do strengthening exercise and aerobic exercise in alternative days from Monday to Friday; however, actually we cannot find proper time every weekday. Sometimes it’s difficult to make an agreement with the client (exercise partner) as we have a totally different timetable and there are lots of assignment to handle at the same time. Therefore, after negotiating with my client, I decide to combine … I will give my verbal instruction through WhatsApp … through Facetime in a more vivid way.’ (G1SA)

Some students actively sought feedback from lecturers in addition to their peers. Reflection and modification of exercise plans followed and the last few procedures repeated themselves until the end of the assessment period.

‘From the experience in week 1, I have to admit that my initial exercise plan could be too demanding. I would make some adjustments to address this problem, including reducing the frequency … I reckon that maintaining flexibility is crucial to favour compliance and enable actual gain from exercises.’ (G2SB)

The procedures identified in the discussion forum fit into the notion of self-regulated learning, where students are in charge of their learning process when they are given a learning goal. This theme suggests that an authentic continuous assessment task can facilitate self-regulated learning. Students take a more active approach and are engaged in their own learning experience, while teachers are the facilitators or consultants when questions arise.

### Questionnaire results

There are 14 items in the questionnaire, six items (Items 1, 3–6, 14) regarding perceived benefits of this authentic continuous assessment; four items (Items 2, 7, 8, 13) addressing perceived usefulness of LMS; and the other four items (Items 9–12) addressing perceived benefits of rubrics. Table 1 and Appendix I show the details of the questionnaire items and results. Ninety out of 117 students (76.9%) completed the questionnaire. All items (except for one) received over 60% positive responses (agree and strongly agree).

Specifically, over 80% of respondents agreed that the exercise prescription assignment allowed them to act as therapists to instruct exercises to others, improved their ability to apply and integrate exercise science knowledge, and enhanced their capability to provide exercise training according to the client’s needs (Table 1). Further, approximately 80% of respondents indicated that feedback from peer and instructors during the assignment period helped them improve their exercise prescription and instruction skills, while the LMS facilitated them to track progress, and access information and feedback at their convenience. Interestingly, item 7, which explored the perceived benefit of the discussion forum on communication, only received 35% positive responses. The finding regarding the discussion forum contradicted the correlation analysis result reported in the next section.

### LMS access data and correlation analysis

Table 2 shows the descriptive statistics of the number of hits and assessment scores from 117 students. The average number of hits on the rubrics page and discussion forum per student were 13.4 and 96.8 over the assessment period, respectively. The number of hits on the timeline of assignment page was the lowest (11.9 times on average). The mean final assessment score was 21.1 out of 25.

**Table 2:** Descriptive statistics of the assessment score and number of hits in content areas in the learning management system (LMS)

	Assessment score (*n* = 117)	Main page hits* (*n* = 117)	Rubrics hits* (*n* = 117)	Timeline of assignment hits* (*n* = 117)	Weekly log hits* (*n* = 117)	Discussion forum hits* (*n* = 117)
Mean + SD	21.13 + 1.90	14.79 + 13.00	13.38 + 9.41	11.94 + 8.79	24.44 + 18.23	96.75 + 118.57
Minimum	12.60	1.00	1.00	0.00	5.00	0.00
Maximum	24.46	81.00	53.00	58.00	128.00	660.00

*Hits = average number of access per student over the assessment period

Spearman’s correlation results showed that the assessment score was significantly correlated with rubric hits (ρ = 0.29, *p* < 0.01), timeline hits (ρ = 0.32, *p* < 0.01), weekly log hits (ρ = 0.34, *p* < 0.01) and discussion forum hits (ρ = 0.59, *p* < 0.01). The correlations between assessment score and rubric hits, timeline hits or weekly log hits were weak, but moderate with the discussion forum hits.

## Discussion

This mixed method study investigated how an authentic continuous assessment coupled with rubrics facilitated physiotherapy students’ learning process in a real-life complex skill of exercise prescription and instruction. The study also explored the relationship between different activities in the LMS and learning outcomes. Qualitative findings from the focus group and discussion forum posts indicated that students used a pattern similar to what has been described in the literature as self-regulated learning. Questionnaire results showed that the LMS facilitated students to track their own progress, and to access information and feedback online. Correlation analysis revealed that students who had more access to discussion forums generally obtained a higher assessment score. Rubrics access, although significantly correlated with assessment score, was found to have the same effects as other content pages of factual information or for submission purpose (e.g. timeline, weekly log). This may imply that rubrics access does not provide additional benefits to assessment score. Nonetheless, to our knowledge, this is the first study to explore the learning effects and process of an authentic continuous assessment in the didactic component of physiotherapy education. Results support the benefits of authentic assessment and aid understanding of the learning process used by physiotherapy students.

Authentic assessment facilitates learners in using essential and complex skills required for real-life situations [6, 8]. Our assessment task design provided such experience because students needed to plan, execute and evaluate their exercise prescription. The process was not a simple linear “coming up with an answer” type but required multiple and cyclical learning strategies, in order to reach the ultimate objective. Results from the focus group and discussion forum analyses revealed that students used cyclical strategies, such as planning, searching, execution, reflection, communication and collaboration in this authentic assessment of exercise prescription. These strategies fit similarly into Zimmerman’s cyclical model of self-directed learning [21]. Planning and searching are activities used in the forethought phase. Execution, communication, collaboration and reflection match the description of the performance and self-reflection phases accordingly.

These strategies were used cyclically by our students. Our students went from planning to executing to reflecting and they planned again. They reflected when things didn’t go according to plan or assumption and this happened quite early on during the exercise period. This may be attributed to the nature of our authentic task. Notably, exercise prescription is not a straightforward and linear process. The exercise instructor needs to constantly adjust the exercise type and dosage, to name a few, according to the physical and psychological responses of the exercising person. The task demand of authentic assessment mimics real-life needs and supports a more cyclical way of self-directed learning. This differs from a simple formative assessment, which may not be continuous.

Rubrics were situated as both a planning and a feedback tool in our study. Similar to previous studies [24], our rubrics offered guidance, increased understanding of expectations and provided feedback (through grade descriptors) to students. These functions are essential for learners to regulate the ways of approaching assessment and, hence, learning within the assessment process. Our rubrics were available from and explained on the first day of class so students could use them to plan the exercise prescription process. We also used a multi-stage design where students received feedback at two time points and a grade at midway to facilitate improvement in the second half of the assessment. Students valued the midway grade and used it to self-assess if they had been on the right track. Effective feedback is a strategy to scale-up AfL [8]. Self-assessment through the use of feedback is also a crucial element to support self-regulated learning [15]. After students had self-assessed and reflected on feedback and the midway grade, the process of planning and doing started again. The way we situated the rubrics enhanced the feedback mechanism and facilitated a cyclical self-regulated learning paradigm as discussed above.

It is however important to note that students in our study appeared to link their learning and self-regulation with their goals of achieving higher grades. It is beyond the scope of this study to investigate if a goal of achieving a higher grade is facilitatory or detrimental to learning. Essentially, it is unlikely to separate the two as assessment serves multiple functions [43] and actualising rubric to its fullest learning function requires a holistic approach of all stakeholders in the education system [29].

It is surprising that the rubrics’ access rate was only weakly (although significantly) correlated with assessment score, and the magnitude of its correlation coefficient was similar to those of other content pages, such as the timeline page (for submission timeline) and the weekly log page (for submission and reading of partners’ posts). This observation might indicate that these contents were accessed for the purpose of checking or submission only. However, the relationship between the rubrics’ access rate and assessment scores might have been affected by the fact that some students downloaded and saved the rubrics file to their computer for the preparation of the assignment. They did not need to revisit the rubrics page every time for referencing as it was more time consuming to login to the LMS. That said, since some students might not have downloaded the rubrics and needed to access the pages for understanding the assessment criteria, there was still a weak correlation between the access rate and the assessment score.

Interestingly, the discussion forum access rate showed the strongest correlation with the assessment score, while students in the focus group reported that the discussion forum did not benefit them in the assessment process. In other words, the students were not aware that accessing the discussion forum did benefit them. The nature of the discussion forum page differed from other pages as the information was constantly updating and also could not be downloaded. Theoretically, motivated students checked and contributed to the forum more frequently. The more they read their peers’ posts, perhaps the more they reflected and self-assessed. Since students could access the forum at any time anywhere, it allowed them to digest and reflect at their own pace which probably facilitated learning [22]. The discussion forum also allowed learners to build upon their ideas, which in itself is an authentic task mimicking the real-life needs of self-directed learning [22, 44]. As such, these benefits might have explained the correlation we found between the discussion forum access rate and the assessment score.

Conversely, students’ scepticism on the workload and authenticity of the discussion forum may affect its perceived usefulness in their learning. In fact, workload has been reported as a students’ dissatisfaction factor of online discussion [45]. These factors may limit our students’ perceptions on the usefulness of the information for self-reflection and improvement. Nonetheless, it is difficult to judge if students’ posts on the discussion forum are real-life encounters or just stories to earn grades [44]. The fact that there was a moderate correlation between the discussion forum access rate and the assessment score proved that learning was happening, possibly through self-assessment and reflection when comparing their own exercise prescription process to their peers, but without conscious awareness of its happening. Explicit explanation of the objectives of the discussion forum, as part of the authentic experience, is warranted for raising cognitive awareness of its benefits [45].

### Limitations

There were several limitations identified with this study. First, it is difficult to determine if this authentic continuous assessment generated better learning outcomes than the previous assessment method that only required the students to document their own exercise habit for 6 weeks. Since the assessment tasks and the rating of each task were different under this new assessment, direct comparison with previous student cohorts is deemed inappropriate. Second, only the LMS access data and the assessment score were used for correlation analysis and they might not represent the full picture of the relationship between learning activities and outcome. To minimize this limitation, we used different sources of data to gain more comprehensive insights from various angles. Third, self-regulated learning was a concept that emerged during the data analysis phase, hence, neither the questionnaire nor the focus group interview addressed this concept. Results might have been richer had this concept been discussed in the interview. Fourth, it was not the aim and interest to investigate the effects on individual assessment activities on learning outcomes. Hence, component effects were not available from this study. In addition, it is also out of this study’s scope to explore the changes in students that might have occurred after this assessment.

## Conclusions

Through redesigning an assessment component of a course into an authentic continuous assessment task, physiotherapy students learnt to apply exercise prescription knowledge and concepts together with a peer. Using rubrics on an LMS as guiding and feedback tools, students utilised cyclical self-regulated learning strategies in this authentic assessment process to guide their learning. The online discussion forum might also have served as an authentic component that supported self-regulated learning, although students did not seem to agree with this benefit. Assessment with an authentic task design and with the use of rubrics as guiding and feedback tools should be promoted in the didactic component of physiotherapy curricula. It is recommended to make objectives of various components (e.g. rubrics, discussion forum) explicit in order to promote use and raise awareness of their beneficial effects. Instead of using the discussion forum as a standard task, teachers should make use of it by designing questions that spark students’ reflection so they can benefit from the process. Future research can also explore metacognitive use of rubrics and discussion forum information in the context of authentic assessment.

## Supplementary information

**Additional file 1.** Results of the Questionnaire.

## Data Availability

The datasets used and/or analysed during the current study are available from the corresponding author on reasonable request.
