# Supplementary material for: Learning to prescribe and instruct exercise in physiotherapy education through authentic continuous assessment and rubrics
Source: BMC Med Educ. 2020 Aug 8;20:258. doi: 10.1186/s12909-020-02163-9 (PMC7414576; doi:10.1186/s12909-020-02163-9)
Supplement: Supplementary file 1 — Additional file 1. Results of the Questionnaire. [file 12909_2020_2163_MOESM1_ESM.docx]

**Appendix I – Results of the Questionnaire**
